# A brief history of FASEB and its programs and activities

**DOI:** 10.1096/fba.2020-00009

**Published:** 2020-06-12

**Authors:** Howard H. Garrison, Judith S. Bond, Ralph A. Bradshaw

**Affiliations:** ^1^ Federation of American Societies for Experimental Biology Bethesda MD USA; ^2^ Department of Biochemistry & Molecular Biology Penn State University College of Medicine Hershey PA USA; ^3^ Department of Physiology & Biophysics School of Medicine University of California Irvine CA USA

## Abstract

The Federation of American Societies for Experimental Biology (FASEB) was formed in 1912 to serve the needs of its four charter societies. Its growth, from these organizations with a little more than 300 members to nearly 30 societies with over 100 000 members, is a tribute to its ability to respond to the changing structure and needs of the experimental biology community. The Federation began as a loosely constructed, single‐purpose organization established to facilitate the coordination of the annual meeting of its four member societies. Following World War II, the limitations of this informal structure became readily apparent, and the development of a professional staff under the leadership of Milton O. Lee ushered in the second phase of FASEB's history. Lee oversaw a period of substantial institutional growth, but when he retired in the mid‐1960s the unresolved issues of governance and member autonomy loomed large. These became increasingly divisive sources of organizational friction and were not meaningfully resolved until the Williamsburg Retreat of 1989 restructured the Federation and initiated the third phase of its existence. The changes made as a result of this pivotal event gave FASEB a new *raison d'etre* (public affairs) and made the organization attractive to many other biomedical research societies. Membership grew rapidly in the 1990s and early years of the 21st century. This larger membership, along with changing financial relationships, present new challenges for the Federation and are precipitating another restructuring.

## 1 INTRODUCTION

**Figure 1:**
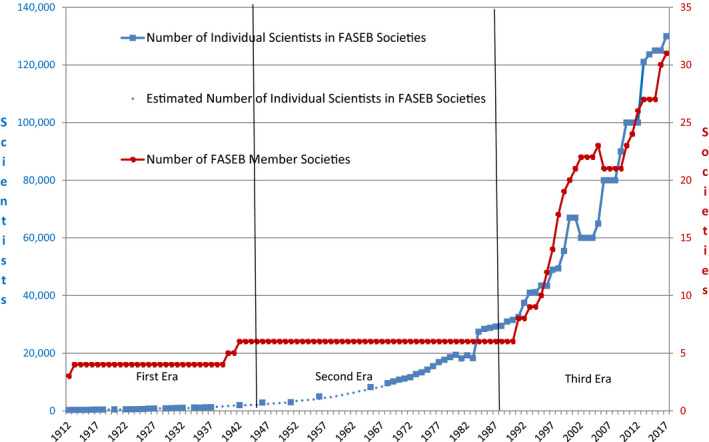
Number of FASEB Member Societies and number of total individual scientists in FASEB Societies, 1912‐2017. Members of multiple Societies are counted only once. Estimated numbers are based on Society counts adjusted for average percentage of multiple memberships or extrapolated from adjacent years

The Federation of American Societies of Experimental Biology (FASEB) consists of nearly 30 academic societies (and their ~125 000 individual scientists and engineers), headquartered in the United States, that are focused on promoting various subdisciplines of biomedicine. Its mission is to “advance health and well‐being by promoting research and education in biological and biomedical sciences through collaborative advocacy and service to our societies and their members.”^*^ The constituent societies range in size from a few hundred members to several thousand, and virtually all have significant numbers of international members. The Federation exists because it has long been appreciated that there are activities that are better confronted collectively, such as advocacy in support of federal funding, dealing with regulatory burden and addressing issues of social/political ramifications, for example, stem cells and animal welfare. It has also been an important source of services that has been particularly valuable to smaller societies, and it has been a respected contributor to scientific communication through its conferences and meetings and its journals. The Federation was not always a major player, starting quite modestly in 1912 in terms of both size and scope and growing unevenly over the 105+ years of its existence. As shown in Figure 1, it experienced three distinct phases of growth and activity that are defined by two major inflection points. On both occasions, it led to changes in the organization that radically altered its structure and enabled it to meet the challenges of the time. It now appears poised to enter its fourth phase. In the following account, the highlights of the history of FASEB are outlined along with the events that shaped the organization as it exists today.

## 2 FOUNDING

The Federation was formed in December 1912 and to a large extent was the formal recognition of previously existing interactions between three societies with substantial common roots and interests. The first of these, the American Physiological Society (APS), was founded in 1887 and it was one of the first American groups interested in biology and biomedical science that emphasized research accomplishments and experimental pursuits as criteria for membership. This distinguished it from broader associations, such as the American Medical Association and the American Association for the Advancement of Science, where membership depended primarily on interest and was open to essentially anyone. This important distinction would remain a fundamental theme that has characterized the societies associated with the Federation throughout its lifetime (and is reflected in its chosen name).

APS remained quite modest in size until the turn of the century when interest in the more molecular aspects of physiology began to grow and research in, and the teaching of, physiological chemistry became more prominent in America.^1^ At this juncture, there were two means for communicating new information in science: publication in an academic journal and presentation at a meeting. The latter, of course, offered much greater opportunities for discussion and analysis and not surprisingly became the focus that led to the formation of the other two founding societies of FASEB—the American Society of Biological Chemists (ASBC)^†^ and The American Society for Pharmacology and Experimental Therapeutics (ASPET). Over time, the pressure to find sufficient place on the program of the annual meeting of APS to accommodate the growing needs of the “chemists” became a real challenge.

This situation, which had been the topic of considerable discussion in the community at large, came to a head in the fall of 1906 when John Jacob Abel of Johns Hopkins University sent a letter with an enclosed circular to the members of the editorial board of the *Journal of Biological Chemistry* (*JBC*) asking for support of a proposal to create “… an association…[for] all who are interested in the biological sciences from the chemical point of view”.^2^ He and Christian Herter had founded the *JBC* the year before and the two dozen members of its editorial board were the logical group to champion a new chemically orientated society. Most, including Abel, were already members of APS, and an alternative solution that had been widely considered was to create a subdivision of APS for such a group. However, Abel argued that the field of biochemistry was broader than that associated with physiology and indeed when the ASBC formed a few months later, this was reflected in the charter membership.^2^

The ASBC was formally created in late December 1906 at a get‐together of 29 individuals held during the APS annual meeting of that year in New York City. The proposal to create a new society was enthusiastically approved and Henry Chittenden of Yale University, who had had a major role in the founding and early governance of the APS, was elected the first president with Abel chosen as Vice President. In subsequent developments, the number of charter members was increased by some 50 additional people who could not attend the New York meeting, and did, as Abel had predicted, broaden the scope of the society to members working outside what was normally considered to be physiology. Importantly, the relationship of the newly formed ASBC with its “parent” society, APS, did not suffer any significant repercussions and the two societies immediately began to correlate their activities, primarily around the national meeting.

Two years later, Abel again exercised his organizational skills and assembled a similar (although smaller) group for a meeting held at his own laboratory at Johns Hopkins University for the purpose of founding a pharmacology society. As with the ASBC, this was unanimously endorsed, and Abel assumed the first presidency. He also announced at this same meeting the launch of the *Journal of Pharmacology and Experimental Therapeutics*, a name the Society also adopted.^3^ The journal was independent of the Society, as was the case for the *Journal of Physiology* and the *JBC*, and it was several years before each was eventually subsumed by the corresponding society.

**Figure 2:**
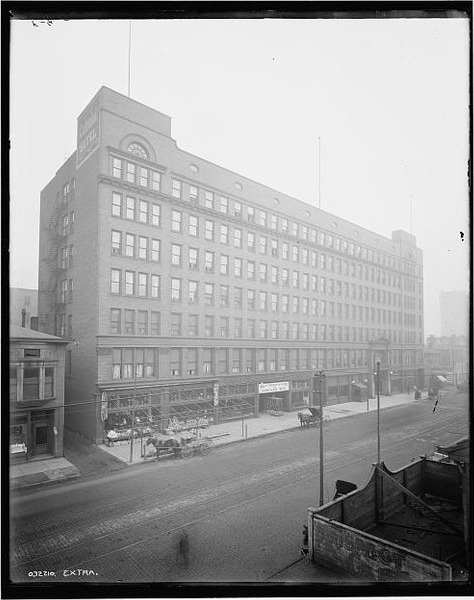
The Colonial Hotel in downtown Cleveland around 1912, site of the Conference Committee meeting that founded the Federation. It is still standing and functioning as a hostelry under the name Residence Inn by Marriott Cleveland Downtown. It was the site of the FASEB 100th FASEB Anniversary dinner and celebration 29 December 2012. Image from FASEB Centennial Website (http://www.faseb.centennial.org/); November 26, 2018)

ASPET immediately joined with ASBC and the APS to plan their annual meetings as joint affairs. Prior to the formation of the new societies, the APS had regularly met with other scientific societies and associations, but this became increasingly more problematic, and the natural affiliation of APS, ASBC, and ASPET (as evidenced by their substantially overlapping memberships) quickly replaced such arrangements. As the memberships of all three societies grew, reaching more than 300 unduplicated members by 1912, their annual meeting became a fixture for scientists pursuing research in these areas, and it only remained to propose and approve the structure that codified the already on‐going interactions and cooperation. This was accomplished through a Conference Committee (with three representatives of each society), which met on 31 December 1912 in Cleveland, OH (Figure 2) and created the Federation by adopting a set of motions that defined it and its activities.^‡^ The Federation was to be run by an Executive Committee composed of the president and secretary of each society with the 1‐year terms of the officers, chair (president), and secretary, determined by a rotation defined by the order the societies were created. The position of secretary to the Executive Committee was of considerable import as the main activity of the new Federation was the creation (and publication) of the program of the annual meeting, which was the responsibility of the secretary.

Although the formation of FASEB gave permanence to an already existing relationship, it is noteworthy that at least some individuals realized that the federation plan offered the potential for much more. A. J. Carlson, the first secretary, in his report on the initial meeting of the Executive Committee, in thoughts that presaged the eventual growth and importance of FASEB, noted:

The distinctive feature of the federation plan is the cooperation and coordination in the essential things with no interference with the individual societies. This cooperation is certainly desirable between the biological societies and we believe the federation plan can and ought to be extended in that direction. We believe it will increase the efficiency of the societies as agencies for the promotion of research and dissemination of truth.^4^

## 3 THREE ERAS AND THE TWO INFLECTION POINTS THAT CREATED THEM

### 3.1 The first era

This initial period spanned the years from the founding of the Federation through the second World War (1912‐1945) and for most of this time involved only the four charter societies. Although it did address a few issues that could be construed as “public affairs,” FASEB primarily functioned as a virtual organization whose purpose was to create, manage, and publish the program of the annual meetings. These Federation meetings were held in the eastern half of the country at various academic institutions that provided a local organizing committee, facilities, and manpower (in the form of students). It was a system that worked remarkably well, given that all these societies were growing in size throughout this period. However, the workload of the Executive Committee, and particularly of the secretary, continued to expand and, as a harbinger of things to come, in 1935, the Bylaws were modified to appoint a permanent secretary, who also became a member of the Executive Committee, the first person not a society officer to do so. The post was filled by Donald R. Hooker, who had been actively involved in the publication activities of APS, and this connection, which continued during his association with FASEB, established a crucial link between APS and the Federation, which was key to the developments following the Second World War.

The end of this era saw two other important events: the addition of two new societies—American Institute of Nutrition (AIN) and the American Association of Immunologists (AAI)^5^—and the launch of *Federation Proceedings*.

Although the members of AIN already had substantial interactions with members of the charter societies creating a natural affiliation, the application of AIN, first tendered in 1933, was not approved until 1940. AAI, founded many years earlier, began meeting with the Federation in 1940 and its application for membership was approved 2 years later. The addition of these two societies occurred just as the Federation entered an enforced hiatus due to wartime restrictions on domestic travel, and there followed a 3‐year period (1942‐1945) when no national meetings were held. Thus, the impact of these new members would not be felt until the start of the second era.

One of the first added functions of the Federation was the creation of a journal. *Federation Proceedings* appeared in 1942 after being discussed by the Executive Committee for several years. The distinguishing feature of the *Proceedings* was the publication of abstracts and symposia articles derived from the national meeting as well as other society contributions.^6^ It also replaced the *Federation Yearbook*, which had begun publication following FASEB's founding*,* and was an annual compendium listing the officers and members of the constituent societies and their committees as well as the Federation officers and its Bylaws. It was incorporated in the *Proceedings* but later (1964) was split out again as the *FASEB Directory*. *Federation Proceedings* was sustained by a per capita assessment of the constituent societies. This mechanism would eventually become a contentious point, and when it increased dramatically it threatened the viability of the Federation.

### 3.2 The First Inflection Point—Lee and the Hawley Estate

The Second World War changed a great number of things not the least of which was a marked increase in interest in science and technology. It is fair to ponder whether this rise in enthusiasm would have occurred anyway, but without the many scientific and engineering advances that were produced by the various war efforts, probably at a much slower pace. In addition, other organizations that had suffered the same inactivity suddenly sprang to life too, the net result of which was that suitable space to hold the annual meeting, now projected to be noticeably bigger, was in short supply. Hooker, just before he became terminally ill, did manage to find a locale in Atlantic City, NJ for the 1946 meeting. However, for the first time, it was not associated with an academic institution, and many of the established features of the pre‐war meetings had to be dropped. This caused expenses to rise sharply and ultimately produced a fiscal crisis, which very nearly ended the Federation following the 1947 meeting when the member societies balked at further assessments and leaned toward closing down the Federation instead. A. Baird Hastings, the Executive Committee Chair, refused to let the committee vote on that decision, and his stubborn support for keeping FASEB alive probably prevented its dissolution at this juncture.

**Figure 3:**
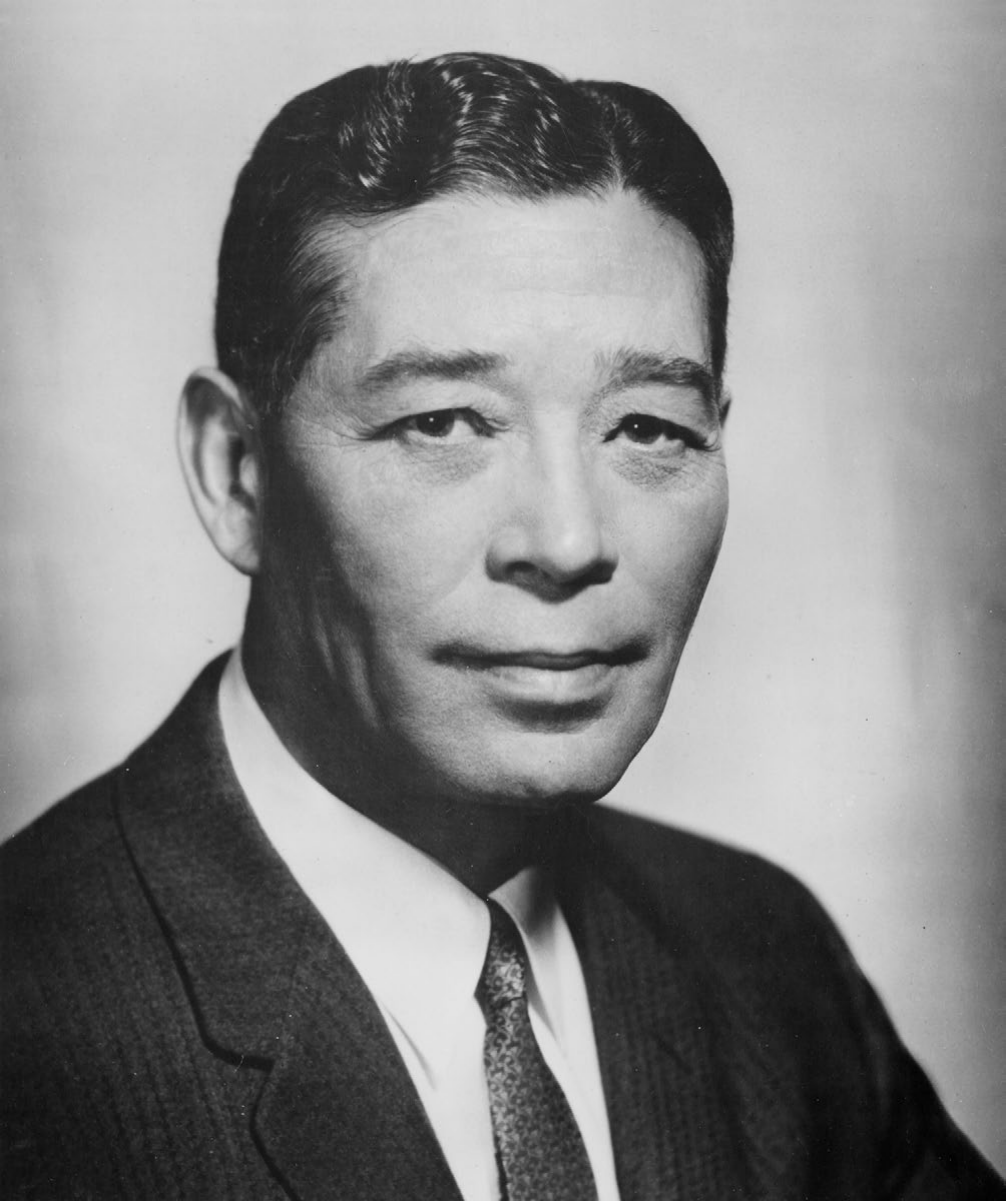
Milton O. Lee (1901‐1978), circa 1947

**Figure 4:**
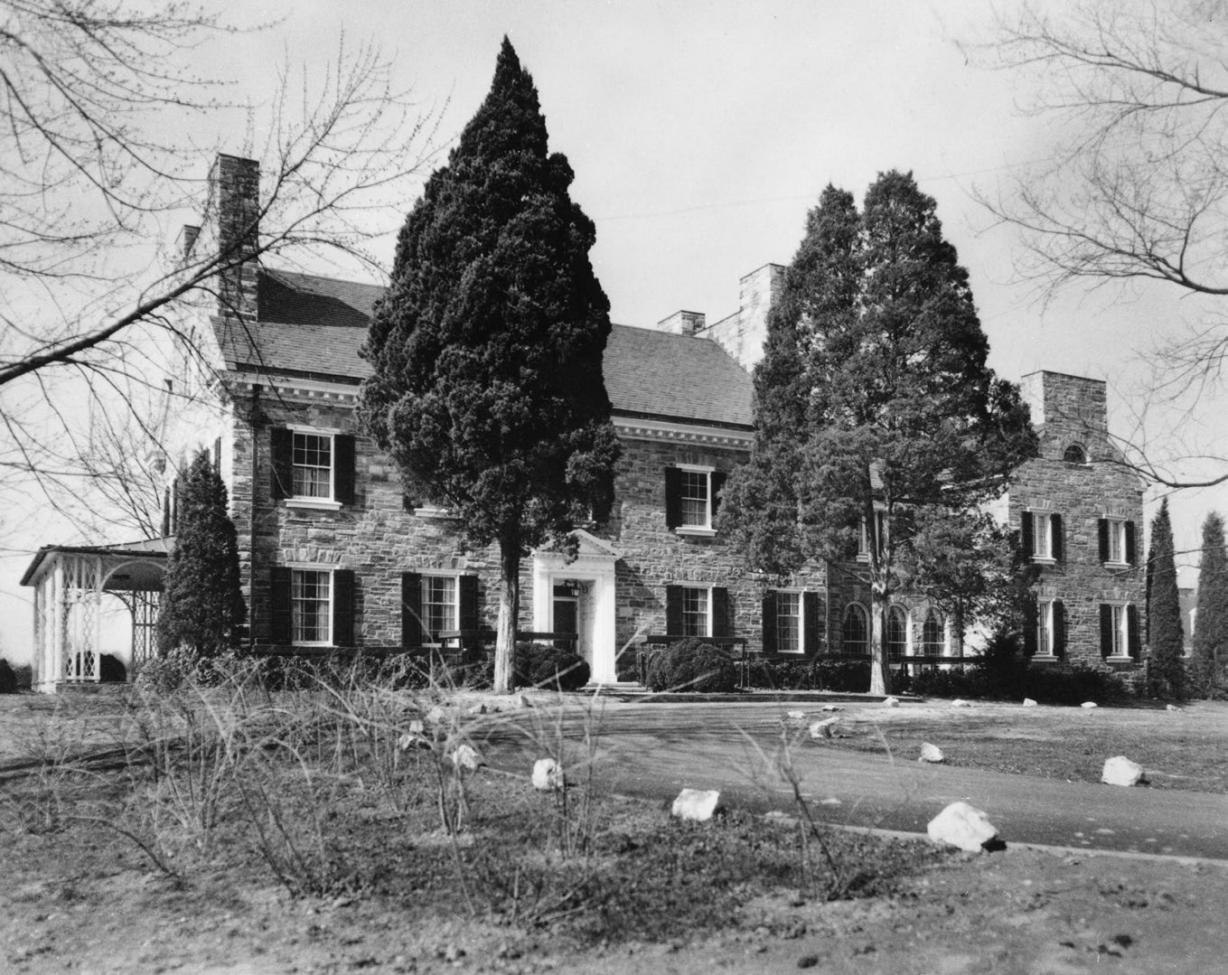
The Hawley mansion in Bethesda was purchased through the combined efforts of the Federation and the American Physiological Society in 1954

It was the APS that suggested that its editorial office be combined with that of the Executive Secretary^§^ and that Milton O. Lee (Figure 3), the APS Executive Secretary and Managing Editor, be also hired as Executive Director of the Federation, responsible for the annual meeting and *Federation Proceedings*. Placing the Federation in Lee's hands was an inspired decision. He took control of the national meeting and introduced substantial changes, such as establishing commercial exhibits, that soon had them operating in the black again. He also established the first real Federation office. Hooker had managed the *Proceedings* out of his own home in Baltimore, and following his death, it had moved briefly to the Medical and Chirurgical Faculty of Maryland. In the fall of 1947, Lee moved both the FASEB and APS operations to space in the National Academy of Sciences (NAS) headquarters, reportedly a corridor, and then, 4 years later, to the NAS Annex at Dupont Circle. The latter move, however, introduced a rental charge ($12,000/year), and the APS began seriously looking for a different solution to alleviate that expense. The Federation, while not opposed in principle to establishing a permanent home, was less active in this pursuit, being mired at the time with governance issues. In 1953, after exhausting possibilities in D.C. proper, Lee and William F. Hamilton (a member of the APS publications committee as well as the committee APS had formed to look for new space) learned that the Hawley estate on Wisconsin Avenue in Bethesda (near the National Institutes of Health) was on the market (Figure 4). In late, 1953, APS negotiated a deal for its purchase, but then had to petition the county for a zoning change, which was not approved until the following summer. Although it was obtained as a permanent home for the Federation, most of the initial purchase price was paid from the APS publication reserves. After the sale was finalized, portions of the 38‐acre estate were sold to a developer (for residential development) and the State of Maryland (for highway easement). The Federation ultimately paid the APS $100 000 for the 11 acres and the buildings thereon that became the Federation home. It first occupied the property in August of 1954.

The Hawley estate consisted of a large home, which was renamed “Beaumont House,” as well as several outbuildings. In addition to FASEB, APS, ASBC, and ASPET all took up residence in the first few years, and it quickly became clear that space would soon be at a premium. Thus, the FASEB Board authorized the building of additional office space, which was completed in 1962. The new building, subsequently named the Lee Building, would undergo several more additions. In 1965, a research wing was added to the east end and in 1967 a matching west wing was built. In 1987 a major additional wing greatly expanded the available space and finally in 2003 the so‐called East Wing completed the Lee Building.^❡^

Although it took several years to complete the transition from a virtual organization to a professionally run Federation with a permanent home and an agenda that reached well beyond the management of the national meeting, the hiring of Lee (and the continued strong ties to APS that it produced) was a “game changer” for the Federation that substantially converted FASEB into a new entity.

### 3.3 The Second Era

The second phase of FASEB's history spanned more than 40 years (1946‐1989), beginning with the lifting of the war‐related curtailments and the hiring of Lee and ending with a major restructuring plan formulated by society representatives. In essence, the Federation evolved into a new organization with changes in how the national meeting would be run, the expansion of its activities and interests, and a stable home office that did not rotate as the volunteer leadership changed annually. During this period, the “new” FASEB that emerged from Lee's 18‐year tenure at the helm (1947‐1965) grew dramatically, reflecting the exciting research findings that characterized the post‐war period and the ever‐increasing investment from both public and private funds. During this period, biomedical research began to attract broad interest and participation, giving rise to regulatory issues that required discussion and management. As the “new” FASEB took shape, Lee had a vision that the Federation would become “the voice of biology.” While Lee oversaw a major expansion of the Federation's facilities and scope of engagement, to some degree his plans were frustrated in this regard by the governance structure of FASEB that did not change significantly. The Federation was created with the notion that there were issues that were better addressed by a coordinated effort but, as the second era unfolded, it became clear that the dividing line between what was a society issue and what was a FASEB issue was not easy to define (or to be agreed upon). In many respects this mirrored the issues confronting a young post‐revolutionary United States when it struggled over how much power the newly formed Federal government should have and what should remain the province of the states. It is fair to say that with respect to the Federation this dilemma was never fully resolved and would frustrate to varying degrees not only Lee but all of his successors. Although he did not fully achieve his goal of making FASEB a “spokes‐entity” in policy and regulatory concerns in the biological sciences, he did create a larger, more visible organization that played a significant role in providing services to the constituent societies and the federal government's growing science establishment.

Lee was succeeded by J. F. A. McManus (1965‐1971), Eugene L. Hess (1971‐1979), and Robert W. Krauss (1979‐1990), who provided the staff leadership during the rest of this second era. This 25‐year period saw substantial institutional growth for FASEB, but it gave rise to critiques, analyses, and dissatisfaction among the member societies regarding the enlarged mission and activities of the Federation. On the upside, the national meeting was dramatically expanded, the Office of Public Affairs (OPA) created and developed, and services/office space for both member and non‐member societies established. In addition, the summer research conferences were created and *FASEB Journal*, a major upgrade of the *Proceedings*, was launched. However, two major reviews in the 1970s of FASEB's organization and services found considerable fault with much of what it did and the inability of the Federation to attract new member societies. A retreat, held in Williamsburg, Virginia, in 1980 had been convened to review the concerns that had been identified in the various review processes and, although reaffirming the value of the Federation, recommended changes in services, public affairs and the journal (*Federation Proceedings*). While these adjustments certainly improved the organization, they did not address the stagnant membership nor the rising costs to the member societies. Many began to express the view that FASEB was usurping more and more of the “biological” agenda from the societies, while they were being asked to foot an increasing part of the bill. When several charter societies began to openly discuss secession, the leadership decided that a second retreat was need.

### 3.4 The second inflection point—The Second Williamsburg Retreat

The 1980s, under the leadership of Bob Krauss, were, by most criteria, very productive ones for FASEB. Indeed, during his tenure, Krauss engineered an upgrade of the journal, started the summer research conferences and greatly expanded public outreach programs among other accomplishments. However, these came at a price in the form of higher dues and there was no expansion of the member societies (these two likely being closely linked). It also did not resolve the fundamental question of whether FASEB should be an actor in its own right or merely provide support to the programs of the member societies. When Krauss announced his retirement in 1990, he argued that there should be^7^

…an appointed President rather than an Executive Director. He [Krauss] felt that if FASEB could attract a “scientist statesman” as the President, the Federation would be on a more even field with the other powerful organizations in Washington such as the AAMC and the AAU. He [Krauss] said that FASEB was the only organization speaking for the individual scientists and that the others spoke for the institutions.

This was not a new idea; it had been espoused by Lee as early as the 1960s, but the societies never liked the idea that another entity (even if they were a part of it) would independently speak for their members. It was, in reality, the resolution of this choice of the future direction of FASEB and its Executive Director—president or manager—that the second Williamsburg retreat addressed. The ultimate decision was to vest authority in the volunteer leadership of the constituent societies.

### 3.5 The Third Era

Implementation of the plans developed at the second Williamsburg retreat brought about the onset of the third era, which was accomplished much more precipitously than the gradual changes in FASEB's second era. Fundamentally, the new plan called for “FASEB to become a decentralized, service‐orientated entity run by elected leaders from the Member societies instead of a centralized operation guided by professional staff”.^8^ The emphasis was on public affairs, substantial dues reductions, and making all other services and activities run on a break‐even basis. It also scrapped the nearly 80‐year process of presidential selection by fixed rotation among the society representatives, replacing it with an election by the Board (made up of representatives appointed by each society). Perhaps the most important change was a mandate to recruit new societies, which was initiated immediately (and quite successfully). Overseeing these changes became the task of Executive Director Michael Jackson, who was selected to replace the retiring Bob Krauss. Jackson, a credentialed scientist, understood the constraints of the executive director position and provided exactly the leadership that the Federation needed going forward into its third era.

This third era lasted about 30 years. During that time, the dictates of the second Williamsburg Retreat to increase both the number of member societies and the public affairs activities were fulfilled in a highly impressive fashion. The American Society of Cell Biology, which had assumed an affiliated status in the late 1980s, became the first new society to join FASEB since before the Second World War, and it was rapidly followed by several others. By the end of the century, the Federation had reached 15 member Societies, and this number would basically double again in the next 20 years. At the same time the advocacy efforts underwent steady expansion. In 1993, FASEB created an office for policy analysis to compliment the on‐going efforts on Capitol Hill (Office of Government Liaison) and 3 years later these were combined to form the OPA under the direction of Howard Garrison. During the post‐Williamsburg era, FASEB's public affairs team created the funding consensus conferences, produced influential documents illustrating the importance of biomedical research, orchestrated and participated in key meetings with members of both the legislative and executive branches (bringing hundreds of scientists to these meetings and forums), and developed improved working relations and interactions with government funding agencies by participating and contributing to discussions on regulation and other policy issues. In the process, FASEB came to be recognized as a key player in all aspects of biomedical science policy and was widely respected as an authoritative voice speaking for the working scientist.

### 3.6 The Fourth Era?

As FASEB started into its second century, its situation, particularly financially, started to change noticeably. Although its programs (public affairs, summer conferences and the journal) all continued to flourish, the demand for services and facilities that it supplied to its members and campus residents shifted dramatically. A building boom followed by the recession of 2008 resulted in a glut of office space in the surrounding area of Montgomery County, and it was not very long before many of the member societies realized that they could lease high quality office space at a reduced cost. The aging facilities of the campus could not compete. In 2015, ASBMB, which had continuously occupied the Beaumont house since 1961, decided to leave the campus and they were soon followed by others. There were additional fiscal issues and problems with the management of the national meeting and, as a result, the Federation found itself in worsening financial straits. In early 2016, a group of Executive Officers challenged the elected leadership to confront these problems. Guy Fogleman, who had been the Executive Director for 10 years, agreed to step down to allow the appointment of an Interim Executive Director (who served for 9 months) and, in 2017, a search committee identified Frank Krause as the new Executive Director of the Federation.^8^

Following the June 2016 board meeting, while the process of identifying a new Executive Director was on‐going, the Federation leadership formed four task forces, made up of all of the members of the Board, who undertook a detailed analysis of the future of the campus, the mission of the Federation, society services, and governance/dues. From these deliberations and subsequent recommendations came the decision to close the campus and sell the property to create an endowment that would help to alleviate the severe fiscal problems that were now facing the Federation. There has also been considerable downsizing of the staff.

Given these changes, it is hard to argue that the campus sale does not mark a new inflection point and that FASEB is about to start its fourth phase. Its public affairs programs, science research conferences, and journals^#^ are all doing well, and several committees and task forces have revamped the governance structure (including revitalizing Board involvement) and reworked the Bylaws, redefined the mission and initiated an active membership drive (with two categories now available). All this augurs well for the future, which is important because the Federation, in cooperation with its professional member societies, plays an essential role at the interface between working biological scientists and the funding entities that support and govern the leading research enterprise for which the United States is recognized worldwide.

## ENDNOTES

*
https://faseb.org/About‐FASEB/Our‐Mission.aspx

† The ASBC changed its name in 1987 to the American Society for Biochemistry and Molecular Biology (ASBMB).

‡ The actions of the Conference Committee were considered legally binding and although each Society Council ratified the agreement during 1913, 1912 is accepted as the founding date of FASEB. During the ratification process, the American Society of Experimental Pathology (ASEP) was formed and its application for membership in the new Federation approved; on this basis it has been accorded charter member status.

§ Hooker stepped down in the spring 1946, and his place was taken by William Chambers who oversaw the meetings in 1946 and 1947. After Lee was hired, he remained as Executive Secretary‐Treasurer until 1948, when he left the post. Lee then took this title and the designation of Executive Director was dropped.

❡ When the Beaumont campus first opened it had only member societies as tenants, but as biomedical science expanded, there were many societies who saw value in the business services that FASEB could provide and took up residence as non‐member societies. After the retreat in 1990 that precipitated the rapid expansion of FASEB itself, these were largely replaced by the new member societies.

# At the beginning of 2019, it launched a second journal, *FASEB BioAdvances*.
